# Notes and Notices

**Published:** 1867-01

**Authors:** 


### NOTES AND NOTICES

The postage on this Journal is twelve cents a year, payable in advance to the Postmaster who delivers it.

Those of our subscribers who failed to receive any number for 1866, will have the same supplied by giving notice; numbers lost or soiled, will be supplied to subscribers for ten cents; to all others, fifteen cents.

Any past number of the Journal from the first month of publication, will be supplied, post-paid, for fifteen cents.

Any subscriber who fails to receive any number for 1867, will be supplied with the same without charge if applied for during the month for which it was published; if later, it must be paid for, price fifteen cents.

Receipts are not sent by mail, because no receipt is needed, as the Journal is not sent to any one unless it is paid for in advance, and the regular receipt of it by mail is proof that it has been paid for by somebody. All subscriptions must begin with January and end with December, as the volume ends with the close of each year. The bound volume for 1866 will soon be ready and will be exchanged for the loose numbers, if in good order, with thirty cents to pay for binding. If the bound volume is desired to be sent by mail send ten cents in addition, or forty cents for binding and postage.

If a person sends a subscription and does not receive a Journal within twenty days, it is because the money has not been received, or the address was not plainly, fully, and correctly given, and it has been sent elsewhere, and notice of this failure to get the Journal from any cause, must be given within twenty days of writing the letter. It sometimes happens that persons complain of not having received their paper at the end of the year, and seem to think they are entitled to all the back numbers, when the cause of non-reception was their failure to give a plain, full address.

We, like others, are often solicited to send our paper without charge to various public and benovolent institutions, associations, libraries, &c.; this is an unreasonable request; it is certainly less burdensome for fifty members to pay three cents each than for one publisher to supply fifty copies of his paper to fifty "institutions" for the bare chance of somebody happening to see it in the "rooms" and be induced to subscribe for it; the 'honor' of having our paper placed on the desk of the great Mogul does not pay for the trouble of sending it to the post-office. Any Association or Society that calculates on begging for a support had better "dry up" incontinently. We are willing to give any quantity of Journals to preachers and Theological students and libraries, but futher than that we do not propose to go, unless we choose to.

A new edition of Hall's Health Tracts, with steel portrait of the Editor, will be issued in January, 1867, price by mail $2.50; contains about 290 Health Tracts.

THE AMERICAN BUREAU FOR LITERARY REFERENCE.—*Agency for Authors, Publishers, Editors, Lecturers, and Lyceums, and for all who have any Literary Commissions to be executed.*

THE COMMISSION undertakes:

1.—To gather facts and statictics upon all subjects, and to present them in an intelligent form, either for literary or business purposes.

2.—To furnish printers' estimates for authors, and to supervise the publication of works.

3.—To receive manuscripts either for sale to a publisher, or to be read for a critical opinion.

4·—To supply translations of books and documents, and to write letters and circulars in various languages; composing the same when desired.

5.—To secure Lecturers for Lyceums and engagements for Lecturers.

6.—To provide suitable editors for newspapers and articles for daily or periodical journals.

7.—To provide correspondents for neswpapers, especially for Washington, New York, Paris, and London.

8.—To select or purchase books for private parties or for Libraries, and to search for rare and old editions.

The Bureau requires a fee of *one dollar* before any Commission is undertaken. The subsequent charges vary in accordance with the actual service rendered.

All communications should be addressed to THE AMERICAN BUREAU FOR LITERARY REFERENCE, No. 132 Nassau St. New York.

Lecturers and Lyceums invtied to put themselves in communication with the Bureau. Charge for entering name, $1.00

BRONCHITIS AND KINDRED DISEASES," with which the January number for 1867 begins is from a book with the same title, sent post-paid for $1.60, by addressing "Hall's Journal of Health," No. 2 West 43d St., New York; the object is to persuade the people to note the first far off symptoms of consumption when the disease can be easily and certainly warded off permanently; and to this end the symptoms of beginning and curable consumption, as well as the indications of a hopeless malady, are so plainly laid down that the most unlettered may determine for themselves the beginnings of danger; it also marks out the difference between Bronchitis, Consumption and Throat Disease by showing the symptoms peculiar to each, and thus the general reader may determine for himself, in markd cases, what is the matter with him

INSTITUTE LECTURES ON PHYSIOLOGY.—*To the Executive Committee of the Institute of Reward for Orphans and Patriots:*

The undersigned, a Committee appointed December 30th, 1865, by the Executive Committee of thc Institute of Reward for Orphans and Patriots to co-operate with the Executors of the Will of the late Miriam Holton Brown, respectfully report:

That the general diffusion of the knowledge of physiological and hygienic laws and their application for the benefit of communties and especially of the rising generation, are to be sought under provisions of the Will, through the continuance of the lectures on Physiology commenced in the city of New York, in 183[illegible], and which for thirty-two years have to some extent been continued by her brother, David P. Holton, M. D., in the public and private schools of Europe and America.

In the futher continuance of these lectures, Dr. Holton desires to labor in those institutions in which his services will be productive of the most good in the establishment of hygienic rules and practice; *and where at the same time the rewards of patriotism can be best advanced in providing for the orphan representatives of those having died or who may die in the service of our country.*

Dr. Holton's selection of physiological topics and their presentation will be determined with a view to the objects above stated, also, to their appropriateness, as means of mental and moral training, securing the three objects—physical, intellectual and moral development.

From his long experience as a teacher, from his mental attainments as a graduate in 1839 of the College of Physicians and Surgeons of the City of New York, and from his subsequent attendance 1854, 1855, 1856, 1857 at the best schools for physiological studies in the Universities of France and Germany, we feel authorized to assume that his selection and presentation will be such as to effect great good.

52 West 37th Street, New York, July 4th, 1866.

HORACE WEBSTER, M. D., LL. D.,

MARSHALL O. ROBERTS,

ALEXANDER KNOX,

SAMUEL B. BELL, D. D.

ARTHUR F. WILLMARTH,

} *Committee.*

THE AMERICAN TRACT SOCIETY, No. 150 Nassau St., New York, have published the "Life and Times of Martin Luther," by W: Carlos Martyn, author of the Life and Times of John Smeton; it aims to continue the biography of Luther and a history of the "Reformation." 12mo., 550 pp. This will be regarded as a standard publication and may be read with interest and profit by christian people; it is admirably adapted to giving even the unlettered a clear idea of what the great "Reformation" means; its bearings on the great religious doctrines of the age and their practical tendencies when carried out in daily life; it is especially valuable to clergymen and students of Church history.

Good Eating.—"Jennie June's American Cook Book," 12mo., 343 pp., is published by the American News Co., 119 Nassau St., New York; it is by the gifted author of "Talks on Woman's Topics," etc. It gives Ruskin's answer to the question, "What does Cookery mean?" and imbodies many of the principles inculculated by Professor Blot (pronounced *Blow,*) and therefore may be regarded as a scientific, practical book, by a woman who has made and done the things herself, and knows whereof she speaks, instead of its being a compilation of impossible and untried things.

Oatman's Fifth Avenue Skating Rink.—*The largest in the world.*

I have the pleasure to announce to my numerous patrons, that I have erected a Skating Rink—the finest in the United States—on the site of the former Balloon Amphitheatre, Fifty-ninth St., corner Sixth-avenue.

This is doubtless the largest Skating Rink in the world, having an area of 7,000 square feet. It is entirely surrounded by a gallery, seated, and covered overhead, with ample space for 10,000 spectators.

Attached to the Rink are handsomely fitted reception and waiting rooms, and a carefully conducted restaurant.

*Ladies are provided with exclusive waiting and dressing rooms.*

The entire Amphitheatre will be lighted during the evening by two hundred gas jets.

The Rink will be the headquarters of the "New York Skating Club," for which ample and special provision has been made.

Music will be in attendance every afternoon and evening, conducted by an accomplished leader.

A selection can be made from the superior stock of skates in the skate-room, and ample provision is made in the cloak-room for the deposit of cloaks, etc.

An important feature of the Rink is that skaters will be at all times protected from uncomfortable wind by the surrounding galleries, which rise above the ice level about 40 feet.

O. F. OATMAN, Proprietor.

*Terms.*—Gentlemen' s Season Tickets................$8.00
Ladies' Season Tickets.................... 5.00
Masters' Season Tickets.................... 5.00

How many men in a thousand in the United States can write their own names sufficiently plain to be read by a stranger, and without any senseless flourishes which, in almost every case, indicates that the writer has no force of character? Query No. Two: How many persons in a million can order a publication and give their name, Post-Office, County and State? Some send no name at all; others omit the state, as if their own little village one rod long and no rods wide, was familiarly known to the utmost bounds of creation. If the reader orders our journal please don't lose time in telling what a useful thing it is everybody knows that; do like the most sensible woman in; the United States—"MARY REED, Dover, Delaware. $1.50 for Hall' Journal of Health for 1867." How delightfully plain, succinct and sensible. She ought to have it for nothing.

Messrs. Broughton & Wyman, 13 Bible House, New York, have sent us a number of little books for a few cents each, which are so good and useful that any parent might send them one, two or a dozen dollars and leave it to their discretion to send the value in these little books for little children, to wit: "No Sect in Heaven," 16 pp. "The Lamb that was Slain," 12 pp. "Self-Examination," 46 pp.—Ten cents. "Social Hints for Young Christians," in three sermons, both by Rev. Howard Crosby, Pastor of the 4th Presbyterian Church, New York; a most admirable issue, in various bindings, 20 to 40 cents.

THE AMERICAN TRACT SOCIETY, 150 Nassau St., New York, S. W. Stebbins, Depository, have issued a number of beautiful Gift Books, and if parents would only spend their money for presents such as these, instead of gew-gaws and jewelry, a life-long good would be the result; such as—

"Jay's Morning Exercises." 8vo. Steel Portrait. $1.75; extra binding, bevelled boards, red edges, $2.75. "Jay's Evening Exercises." 8vo. $1.75; extra, $2.75 "Burder's Village Sermons." 8vo. In clear type. $1.50; gilt, $2.00 "Sketches from Life." First and Second Series. Illustrated. Each $1.10. Extra binding, $1.75 each. "Life of George Whitfield. With Engravings and Steel Portrait. $1.10; extra, $1 75. "Records from the Life of S. V. S. Wilder." With fine steel portraits. A volume of rare interest and value. $1; extra, $1 50; mor. gilt, $3. 50. A book for every son. "Baxter's Saints' Rest." 12mo., large type. $1; extra, $1 50. Also 18mo, extra, $1 "Bunyan's Pilgrim's Progress," with Grace Abounding prefixed, 12mo, finely illustrated, $1 50; gilt, $2; morocco, gilt, $3 50. Also 18mo, extra, $1; gilt, $1 25. "A Pastor's Jottings." Illustrated. Highly interesting facts in a pastor's experience. $1

extra, $1 50. "Eloquent Preachers." Six steel Portraits. Graphic and stirring sketches. $1; extra, $1 50. "Bible Emblems." By Rev. E. E, Seelye of Schenectady, N. Y. 222 pp. square 12mo. Developing the beauty and force of many emblems employed in Scripture, such as the Higher Rock, the Sun in his strength, the Altar of Incense, the Rainbow and the Dove, and applying them to our daily life. The sketches are graphic and rich in instruction. "Its style is almost perfect. It is a beautiful book, and must attract devout readers, old and young."

"Jesus Christ's Alluring Love." 158 pp., 18mo, in fine binding, A rich and attractive devotional manual. "Charles Scott, or, There's Time Eonugh." 147 pp,, square 16mo. 60 cents; postage 12 cents. Life on the sea-shore; the history of an orphau boy, and his battle with a bad habit. "Nuts for Boys to Crack." By Rev. John Todd, D. D. 267 pp., 18mo. Treating a variety of distinct topics in the pointed, shrewd and racy style which makes this author's writings so popular and impressive. He hits the nail on the head, drives it home, and clinches it.

"In the World, not of the World:" being Thoughts on Christian Casuistry, by William Adams, D. D., Pastor of Madison Square Prebyterian Church, New York; a most practical christian book and well worthy of being made a standard publication among all christian people; with such men as Secretaries and Managers as Hallock and Eastman and Stephenson the public have a guarantee that every book issued will be of sterling value and suitable for christians of every name and country.

Messrs. Broughton & Wyman of 13 Bible House, Astor Place, New York, have on hand all the publications of the American Tract Society, Boston; — Uncle Downie's Home; There's Time Enough; Winnie and her Grandfather; The Little Gold Keys;— each 50 cents; Grace's Visit, 75 cents; Madge Graves, $1; Story of Zadoc Hull, 80 cents; Frank's Search for Shells, $1 25; Nellie Newton, 45; Lift a Little, 35; Pleasant Grove, 60.

The Messrs. B. & W. are also the sole Agents in New York City, for the sale of "Massachusetts in the Rebellion," by P. C. Headley, author of "Josephine," &c., containing eight steel plates besides many likenesses of distinguished men, including Gov, Andrews, Senators Wilson and Sumner, Edward Everett, Generals Banks, Butler, Stevenson, &c. Price $4 50 to $6 50. They have also issued a book which, in these times of a growing skepticism, is peculiarly timely, entitled "Tests of Truth," being replies to letters of a skeptical friend, on the Teachings of Nature and Revealed Religion, by David Dyer. If any one sees in himself the slightest indication of a questioning of the Divinity of the Bible, let him, for his own soul's saftey, buy this valuable book.

Among the Holiday issues of the American Tract Society a 28 Cornhill, Boston, and 13 Bible House, New York, gilt-edged and bound iu elegant style, are "Snow Flakes," which surprises the reader, not only with the religious sentiment of the volume, but with the scientific wonders which are brought to light respecting the nature, the forms, and the beauties of the beautiful snow. It will give every attentive reader a new idea of the wonderful wisdom and workmanship of the Great Maker of us all. Its cost is about two dollars, with beautiful illuminated and colored illustrations. Also, the Christian Armor of shield and buckler and breastplate, with their various meanings and uses; also, the Cup Bearer and its fellow, the Standard Bearer. Let all who think that the best knowledge is that which leads to the accurate knowledge of the Holy Scriptures purchase these books for themselves and their friends and children, and it will be a good investment.

Seventeen Editions! — in French, several in London, and one in New York, 12mo, 399 pages, published by the American News Company 121 Nassau street, New York, price $2; sent by mail for same. We do not believe that a more deeply interesting and practical book, adapted to the capacity of all, and useful to every human being, has been published in many years, in reference to human health and life. A man took it up carelessly not long ago, and read it through without stopping, except to eat and drink. Its title is "The History of a Monthful of Bread." It takes in the whole subject of nutrition, from the taking of the food into the fingers until it has answered the great object of sustaining life and health and vigor; it shows in an enticing manner the whole workings of the human machine; we bespeak for it an extraordinary demand, all over the nation. To thoughtful, progressive minds, its perusal will be a delight; but as most persons of this class are in moderate circumstances and may not be able to purchase it, we will send it post-paid to any one sending four new subscribers for 1867.

Robert Carter and Brothers, 530 Broadway, New York, have the most extensive stock of standard religious and theological books in the United States, and have unusual facilities for procuring promptly, the new publications abroad. The publications of this house are invariably of sterling and substantial value, not only for the present time, but for future years; among the issues suitable for holiday presents and for family reading are: "Binding the Sheaves," by the author of 'Win and Wear' series; 416 pp., 12mo. "The Story of Martin Luther," edited by Dr. Whately ; 354 pp., 12mo. "The Great Pilot and his Lessons," by Rev. Richard Newton, D. D., author of 'Rills from the Fountain of Life,' 'The Best Things,' 'Bible Lessons,' &c. 309 pp., 12mo. "Cripple Dan," by Andrew Whitgift ; 330 pp. "A Ray of Light," by the author of 'A Trap to Catch a Sunbeam ;' 158 pp. "The School Girl in France," by Miss R. Mc Crindell, author of 'The Converted.' 248 pp. "Win and Wear," a Story for Boys, and a well-told story, too, of youthful struggles and triumphs. "Tony Stars Legacy, " a veritable boy, neither worse nor better than others, and well-nigh spoiled for a time, but at length developes into an upright and generous manhood. "Faithful and true," being the history of a family, reduced in circumstances, retiring to a deserted farm, standing by itself on the Green Mountains. The experiences of this sort of frontier life are depicted with a a skilful pen. "Ned's Motto ; or, Little by Little." Ned's father having fallen in battle Ned worked his way up to usefulness and respectability ; "it is a tale of uncommon excellence." "My New Home," being the diary of a maiden aunt living in a pastor's family in the mountains of Vermont. A critic says : "We have not read a book in which the lights and shadows of such a life are given, with so much truth and vigor." "Turning a New Leaf," being a picture of School Life, with its temptations and social influences, its duties and its dangers. The spoiled child turns over a new leaf, and in the end commands the reader's sympathy and respect.

Fowler & Wells have published a useful almanac for 1867, price twenty cents, being an illustrated annual of Phrenology and Physiognomy, with a multitude of illustrations. The same house has issued one of the most beautiful editions of Æsop's Fables, on tinted paper, gilt-edged, &c., we have yet seen. We do not know of any book, as a present for children, which is better calculated to impress wise lessons of life on the minds of the young—lessons of human nature, which, if early learned, will have a saving influence on all the after life.

A New Monthly, published by the American Tract Society, 28 Cornhill, Boston, and 13 Bible House, New York, being an illustrated religious magazine for the family ; — Vol. 1, No. 1, Jan., 1867, $2 a year, 67 pages. The nature and character of this new candidate for public favor will be best known by the subjects treated, and their authors : "The Sabbath at Home," by Rev. E. N. Kirk, D. D. The new "Morning Star," with four illustrations. "Mary Lyon"

First School Teaching," by Fidelia Fisk. "The Catacombs of Rome," with ten illustrations. "The Battle of Ristori," by Mrs. Helen E. Brown. "The Electric Telegraph," from the British Workingman. "Welcome to a Young Pastor" by S. F. Smith, D. D. "The Parable of the Good Samaritan," by Dr. Guthrie. "George N. Briggs," with a portrait. "The One Thing Needful," from the Sunday Magazine. "The Glory in the Cloud," by Rev. H. M. Dexter, D. D. "The Old English of our Bible," by A. E. "An Appeal in behalf of the Little Ones," by a Mother, etc., etc. No doubt this magazine will contain safe and instructive reading, always, for christian families; and as far as it tends to exclude secular newspapers and secular monthlies from families on the Sabbath, without diminishing the interest and practice of Bible Reading, we certainly wish for it the most abundant success; and trust it will grow in public christian favor with each issue, because of its substantial value.

A woman who is a soldier, as to battling bravely with life's difficulties, writes, Dec. 11, 1866, supposing she was becoming dropsical from the extraordinary bloating of the skin, and fearing it might result in dropsy of the lungs, "The bloating is very little, sometimes none at all, which has not been the case for a whole year, while the regularity of the system is better than it has been for two years; daily improving and growing stronger; it seems perfectly delightful, scarcely natural after so long a time of disturbance. There is a marked change since I applied to you six weeks ago; I think that but for you I should not have lived five months. Others notice the change in me; I am so happy. I find such a quantity of concentrated food for the mind in the volume of Health Tracts; it is not to be digested in a hurry; since reading it I have been astonished and chagrined at my ignorance of so much which is of such vital importance for every person to know."

It is to be regretted that so much indifference exists among all classes as to the means of preserving health and maintaining a good constitution. But as the multitude pay no efficient attention to religion till death is threatened, so but few, here and there one or two in a hundred, feel the inestimable value of health till it is lost, and a once noble constitution is irrevocably shattered. This Journal is devoted to one object, and that is, to show the people how to keep well; how to preserve the body in the enjoyment of glorious good health. If you are sick go to an educated physician in your own community and do not make fools of yourselves by sending money to strangers, who will engage to cure you of everything but the malady of a "soft head;" it would'nt be profitable for them to undertake that, it is because of that they find their enormous gains, by means of which they live in the finest houses on 'The Avenue.'

To PHYSICIANS.—The entire profession will be glad to learn that Henry C. Lea, of Philadelphia, will resusticate the re-publication of "Rankin's Half-Yearly Abstract of the Medical Sciences," during 1867, discontinued six years since by Lindsey & Blakinston. It will be, as before, a synopsis of Medical Progress throughout the world for each preceeding six months. It will be sent to subscribers, free of postage, for $2 50 a year; the Medical News and Library, $1 a year; The American Journal of Medical Sciences, $4 a year. But the three publications, the Journal, News and Abstract, will be sent to one address, post-paid, for $6 00, in advance.

### SKATING.

Private ponds were opened for skaters Dec. 12, 1866; that on the corner of 5th Avenue and 59th St.—A. McMillan, the Prince of Skaters, Manager—is the largest in the city and is accessible by almost every line of cars, and from all parts of the island; it posesses one very great advantage and comfort—you reach comfortably warmed rooms in three or four steps above the ice; and music and mirth are promised every evening that the ponds are open for skating. Up to this present writing it is the best, smoothest and strongest ice in the city, and every pains will be taken to make every day of the winter a skating day.

A Season Ticket for a gentleman is eight dollars; for ladies, five dollars; children, four dollars. Tickets for a single admission. fifty cents.

MacMillan is the sole agent for New York Club Skates, at 575 Broadway, New York, where will also be found a general assortment of fine skates, and Brook's skating boots.

---

No receipt is given for the Journal, as it is only sent to those who have paid for it; its regular receipt is proof of payment.

---

### GOOD BOOKS FOR PRESENTS.

**New Physiognomy,** with 1,000 illustrations, $5, $8 or $10. It is a beautiful book.

**Æsop's Fables,** People's pictorial edition, tinted paper, only $1.

**Illustrated Family Gymnasium.** $1 75.

**How to Write, How to Talk, How to Behave, and How to Do Business,** in one volume, $2 25.

**Tha Phrenological Journal for 1867,** only $2.

Address FOWLER AND WELLS, 389 Broadway, New York.

A brother doctor writes, "I find it an exceedingly easy matter to get subscribers to Hall's Journal of Health, and had I more time to devote to it, I could double the number of names I send you (24) in our village, in a short time."

A correspondent says: "The Journal of Health I must have, if I have to go into the harvest field to work to earn the money. I wish every family in the land had it and would put its teachings into practice; it has been of great value to us. I have tried the receipts in the December number on Winter Diseases, &c., for preserving shoes, giving them a gloss, keeping the feet warm, and it gave great satisfaction, and so with the others."

——VALUABLE information is found in the number for December, 1866, in relation to the preservation of the health in winter-time—Pneumonia, lung fever, inflammation of the lungs; death-in-Doors; how Clergymen lose their voice; airing chambers; temperature of rooms; value of complaining, and crying; getting chilly; having nothing to do: its pernicious effect on health; the bad effects on mind, morals and body of boarding-house and hotel life; how the young should go to housekeeping; getting married; why young men don't propose now-a-days; helping parents; how to make new shoes fit; how to prevent squeaking shoes; how to make shoes impervious to water; varnish for shoes; to prevent cold feet when traveling; to prevent burning feet; tight shoes; cleaning shoes; fruitful source of colds during winter; — sent post-paid for fifteen cents. Address "Hall's Journal of Health, No. 2 West 43d St., New York."

The most generally valuable book we have published is "Health and Disease," $1 60, showing how to avoid sickness and how to cure it in many cases by diet, exercise, etc.

---

### INVALIDS GOING SOUTH.

"AIKIN HOTEL," having been recently renovated and refurnished, is now open for the reception of visitors. Guests can rely on every exertion being made to render them comfortable and make them feel at home. The elevated situation of Aikin, with its dry, equable and genial climate, is peculiarly adapted to invalids affected with pulmonary diseases, and is highly recommended by eminent physicians, North and South.

HENRY SMEYSER, Proprietor.

AIKIN, South Carolina, Dec. 1, 1866.

